# Supplementary material for: Screening and targeted sequencing of stool for microbiologic confirmation and drug resistance determination in paucibacillary tuberculosis
Source: PLOS Glob Public Health. 2025 Nov 21;5(11):e0005243. doi: 10.1371/journal.pgph.0005243 (PMC12637971; doi:10.1371/journal.pgph.0005243)
Supplement: Supplementary file 1 [file pgph.0005243.s001.docx]

Specimen #	pDST	XP-Sputum	Xp-Stool	Nanopore tNGS stool	Deeplex tNGS stool
1					
2					
3					
4					
5					
6					
7					
8					
9					
10					
11					
12					
13					
14					
15					
16					
17					
18					
19					
20					
21					

Rifampicin

Neg	8	2	4	4	7
Ind	1	2		3	1
Sensitive	11	15	10	9	10
Resis	1	2	1	4	2
No results			6		1
