# Supplementary material for: Screening and targeted sequencing of stool for microbiologic confirmation and drug resistance determination in paucibacillary tuberculosis
Source: PLOS Glob Public Health. 2025 Nov 21;5(11):e0005243. doi: 10.1371/journal.pgph.0005243 (PMC12637971; doi:10.1371/journal.pgph.0005243)
Supplement: Attachment — Submitted filename: Response to Reviewers_June7.docx [file pgph.0005243.s002.docx]

Response to Reviewers

**Thank you so much for your invaluable feedback. We have integrated your suggestions and feel that the manuscript is much stronger. We have provided a detailed response to each of the comments below in bold.**

1. Please remove "Brief Report" from the title of your manuscript. Please update it in the manuscript file and online submission information.

**Thank you; this has been done.**

2. In the online submission form, you indicated that your data will be submitted to a repository upon acceptance. We strongly recommend all authors deposit their data before acceptance, as the process can be lengthy and hold up publication timelines. Please note that, though access restrictions are acceptable now, your entire minimal dataset will need to be made freely accessible if your manuscript is accepted for publication. This policy applies to all data except where public deposition would breach compliance with the protocol approved by your research ethics board. If you are unable to adhere to our open data policy, please kindly revise your statement to explain your reasoning and we will seek the editor's input on an exemption.

**Thank you, we have submitted the sequencing data to the sequence read archive (SRA) under submission number SUB15320068.**

3. Please provide separate figure files in .tif or .eps format.

**Thank you, the figure has been updated to be a .tif file and has been removed from the main body of the manuscript.**

4. We do not publish any copyright or trademark symbols that usually accompany proprietary names, eg (R), (C), or TM  (e.g. next to drug or reagent names). Please remove all instances of trademark/copyright symbols throughout the text, including ® on page 4, 5.

**Thank you; these have been removed.**

5. Your manuscript is missing the following sections: Introduction. Please ensure these are present, and in the correct order, and that any references to subheadings in your main text are correct. An outline of the required sections can be consulted in our submission guidelines here:

https://journals.plos.org/globalpublichealth/s/submission-guidelines#loc-parts-of-a-submission

**Thank you; we have updated this.**

Reviewer #1: I have reviewed the manuscript by Ness et al entitled "Screening and Targeted Sequencing of Stool for Microbiologic Confirmation and Drug Resistance Determination in Paucibacillary Tuberculosis (Brief Report)". This is an informative manuscript in an area less researched. I have highlighted areas for improvement below:

1) The abstract is too brief and seems not to be representing the entire manuscript.

**Thank you, we have expanded the abstract to a more standard manuscript length (previously it was formatted for a “brief report”) and now is more encompassing of the entire manuscript, including background, methods, results, and conclusion.**

2) Background: The burden of TB and diagnostic challenges as per the study setting and country are missing. The 2nd sentence under background is too long, please consider revising the writing style throughout the manuscript.

**Thank you, we have added a sentence describing the burden of tuberculosis specific to the study setting with the following:**

***Eswatini continues to be one of the highest burdened countries by tuberculosis, with a tuberculosis incidence of 350 per 100,000 and multi-drug or rifampin resistant TB (MDR/RR-TB) incidence of 18 per 100,000.^1^***

**We have broken up the second sentence into two sentences per your recommendation to ensure clarity.**

***Targeted next generation sequencing (tNGS) has shown to improve the diagnosis of tuberculosis^2^ when applied on samples collected at the point-of-care (including direct sputum^3^ and stool^4^). Targeted NGS can extend the identification of mutations conferring resistance to antibiotics used to treat tuberculosis, including antibiotics for which phenotypic drug susceptibility (pDST) is not commonly available in high-burden settings.^3^***

3) Thirty to 60% of individuals affected by pulmonary tuberculosis... Is its affected or infected, better to use the term "presumptive TB patients"

**Thank you for pointing this out. We try to avoid the word “infected” since that is often used by individuals to describe latent tuberculosis “infection”. We have revised the text to say *“Thirty to 60% of individuals with pulmonary tuberculosis disease”* as this text is specifically referring to individuals who have been diagnosed with pulmonary tuberculosis.**

4) Under last part of background: "we performed a prospective diagnostic accuracy study with participants with undetectable AFB " please revise ..accuracy study "AMONG" participants . Also, the term "undetectable AFB" is confusing, please use smear negative or negative for the primary test.

**Thank you. We have updated this to say *“…we performed a prospective diagnostic accuracy study among participants with undetectable AFB on sputum microscopy (“smear negative”) in Eswatini, a tuberculosis high-burden country”* and used “smear negative” throughout the manuscript to ensure clarity.**

5) Under methods section "Data was accessed for analysis January 2025., very brief sentence and not clear, please revise

**Thank you, we have revised this to “Existing patient data was accessed for this analysis in January 2025.”**

6) ....Foundation Eswatini in Mbabane, Eswatini, and continue to participate in longitudinal follow-up, This is not clear, please revise

**Thank you, we have clarified this to *“…and continue to participate in longitudinal follow-up for capture of treatment and clinical outcomes.”***

7) ..Pulmonary tuberculosis was defined by a) detection of M. tuberculosis in sputum, and/or b) detection of M. tuberculosis DNA by Xpert Ultra on sputum or stool, and/or c) detection of lipoarabinomannan [LAM] in urine in PWH. Please consider making this section clearer 1) define all terms used in standard way, including undetectable AFB, if you choose to maintain it.

**Thank you, we have clarified this section with the following:**

**Confirmed pulmonary tuberculosis disease was defined by signs/symptoms of tuberculosis along with any of the following microbiologic confirmation consistent with in-country testing and clinical standards:**

**a) detection of *M. tuberculosis* in sputum via Xpert Ultra or sputum culture,**

**b) detection of lipoarabinomannan [LAM] in urine in PWH,**

**c) detection of *M. tuberculosis* in stool via Xpert Ultra in children under 15 years of age (reference added) or quantitative polymerase chain reaction (research use only).**

**We have gone through the manuscript to ensure undetectable AFB is clearly understood. In the Introduction, it now states:**

***“Thirty to 60% of individuals with pulmonary tuberculosis disease are smear-negative (have undetectable acid-fast bacilli (AFB) on sputum microscopy),^5^ defined as paucibacillary disease”***

**and *“with undetectable AFB on sputum microscopy (“smear negative”)”***

**In the Methods section it now clarifies:**

***“Individuals were included in the analysis if they were diagnosed with pulmonary tuberculosis disease and had a negative sputum smear (undetectable AFB smear/paucibacillary tuberculosis).”***

2) LAM among PLHIV, this is a conditional test and it should not be used uniformly but rather guided by CD4 and whether the patient was admitted,

**Thank you for highlighting this. Based on the updated guidance from the World Health Organization, it is appropriate to use LAM in outpatient settings regardless of the CD4 count if they have signs/symptoms of TB. This is the standard approach in Eswatini that is followed and we have included the updated in-country guidelines as a reference in the manuscript**

<http://swaziaidsprogram.org/wp-content/uploads/2024/08/Eswatini-Integrated-HIV-Management-Guidelines-2.pdf>

<https://iris.who.int/bitstream/handle/10665/381003/9789240107984-eng.pdf?sequence=1>

**“In outpatient settings**, **WHO suggests using LF-LAM to assist in the diagnosis of active TB in HIV-positive adults, adolescents and children: with signs and symptoms of TB (pulmonary and/or extrapulmonary) or seriously ill; or else irrespective of signs and symptoms of TB and with a CD4 cell count of less than 100 cells/mm3.”**

**https://www.who.int/news/item/04-11-2019-who-updates-policy-for-the-use-of-lateral-flow-urine-lipoarabinomannan-assay-(lf-lam)**

3) Is stool GeneXpert test a standard test for TB diagnosis among adult PLHIV presumptive TB patients in Eswatini? if not, it can not be used in the definition of a case in such population

**Thank you for this inquiry. We have amended this section per above to be more clear and included a citation to the current recommendations.**

4) Please elaborate more on the data analysis and possible adding a sub section to that.

**Thank you, we have done this to ensure clarity.**

5) please define the "paucibacillary' nature of this study population

**Thank you for pointing this out. We have gone through the manuscript and updated this to ensure clarity. In the Introduction and Methods it now defines paucibacillary as “smear-negative” or “undetectable AFB” on sputum microscopy.**

8) Please consider more sub sections as required by the journal under the methods section.

**Thank you, we have updated the Methods section with several subsections to ensure clear organization.**

9) Under Results section: Participant ages ranged from 0 to 68 years (median 32 years).. Please consider presenting median age ( IQR) in a standard way

**Thank you, we have changed this to** *“32 years (rang 0 to 68 years)”* per your request.

10) Throughout the results section, please add percentages to all proportions.

**Thank you, we have added this per your request.**

11) "Targeted NGS on stool provided complete or near complete drug resistance predictions"...What do you mean by "prediction "consider using "detection "or define what you mean by prediction under methods section.

**Thank you for asking about this. It’s standard nomenclature to state “prediction” or “association” for sequencing results for drug resistance. This is the language used by the WHO and others in the field so we will leave it unchanged at this time but are open to changing it if all reviewers are in agreement this is a necessary change to make.**

<https://www.who.int/publications/i/item/9789240082410>

<https://genomemedicine.biomedcentral.com/articles/10.1186/s13073-025-01458-0>

12) Under discussion" Targeted NGS using Deeplex and nanopore provided increased case confirmation of 14.1% and 18.8%, respectively, and full drug resistance predictions in 11.8% and 14%, respectively. Please discuss the possible explanations of this difference in detection rates between Nanopore and Deeplex.

**Thank you for pointing this out, we have added *“The differences in detection between the two commercial kits could be due to differences in sequencing technology (nanopores detecting electrical currents compared to sequencing by synthesis) having different sensitivity rates for M. tuberculosis DNA in stool samples. Further research studies are needed comparing these two commercial kits on extracted stool DNA samples to delineate further.”***

13) "Further, their study excluded children and included few (14%) participants with HIV,7 which are important populations who stand to benefit from tNGS to predict M. tuberculosis drug susceptibility and resistance from stool" What is the difference between drug susceptibility and resistance? please revise.

**Drug susceptibility means that tuberculosis is susceptible to, or will be effectively treated with, the particular antibiotic drug. Resistance is when the tuberculosis will not be effectively treated by the particular antibiotic drug. We have removed “*susceptibility and*” from this to ensure clarity.**

14) In conclusion, application of both commercially available tNGS assays to stool of participants with paucibacillary tuberculosis" Please mention the tNGS assays since they are just two i.e. Nanopore and Deeplex.

**Thank you, we have added this to the section per your request. We would like the editor to confirm that this is acceptable to the journal as we generally limit reference to the commercial assays.**

Reviewer #2: Summary
Ness and colleagues have investigated the use of 2 commercially available tNGS assays, Deeplex and Nanopore, on stool samples from 21 participants with TB. Study participants included children and adults and all were AFB smear negative. This brief report provides some evidence for the utility of tNGS on stool samples, which is a promising finding as people with TB who cannot undergo traditional DST methods can still benefit from receiving Mtb confirmation and drug resistance testing. Some specific comments are provided below, particularly to improve clarity and consistency of reporting.

Specific comments
Abstract: I imagine the word count for a brief report abstract is very tight, but as it stands the abstract does not provide enough information to know what happened in the project. Could the abstract be revised to include the study objective, test/samples, and participant population? Something similar to the stated objective at the end of the introduction would work well.

**Thank you for this comment. We have expanded the abstract to include these details.**

Additionally, the meaning of the phrase “…can detect Mtb DNA in 14-19%” is unclear. 14-19% of what? All patients with TB? All patients with paucibacillary TB? all tested patients? Please clarify in-text.

**Thank you- we have ensured the phrase contains *“of patients with paucibacillary tuberculosis”* to provide clarity to these percentages.**

Methods: Description of lab testing procedures is quite clear. Could you please clarify if all lab procedures and index test were run on-site in Mbabane?

**Thank you for bringing this up. We have added *“All laboratory testing procedures were done on-site in Eswatini.”***

Methods: Can you please provide further details regarding how participants were recruited for this particular study? Were all participants known to have TB before being recruited into the study? Were there any participants who did not have TB who were included in the study? Or was it a presumptive series of people seeking care? Something else?

**Thank you, we have clarified this in the methods section with *“All participants in this study were diagnosed with pulmonary tuberculosis per standard in-country testing.”***

Methods: As written, it is a bit difficult to understand who this project’s participants are. Please include details such as the inclusion and exclusion criteria, e.g., age, HIV status, smear status. How was paucibacillary/undetectable TB defined? Did all 21 people meet this definition?

**Thank you for pointing this out. We have included the following in Methods to make this clearer: “*Individuals were included in the analysis if they were diagnosed with pulmonary tuberculosis disease and had a negative sputum smear (undetectable AFB smear/paucibacillary tuberculosis).”***

**In addition, in Methods we have revised it to state *“From 250 participants with pulmonary tuberculosis diagnosed in Eswatini during our study period, 85 (34%) had a negative sputum smear. Of these, 21/85 (24.7%) participants had adequate M. tuberculosis DNA shed in their stool for tNGS per previous studies and underwent sequencing.”***

Results: among the 8/21 PTB+ individuals who were sputum culture negative, how was TB diagnosed?

**This is now clarified under our Methods with, *“Confirmed pulmonary tuberculosis disease was defined by signs/symptoms of tuberculosis along with any of the following microbiologic confirmation consistent with in-country testing and clinical standards:***

***a) detection of M. tuberculosis in sputum via Xpert Ultra or sputum culture,***

***b) detection of lipoarabinomannan [LAM] in urine in PWH,***

***c) detection of M. tuberculosis in stool via Xpert Ultra or quantitative polymerase chain reaction”***

Results: The phrase “…providing a 14.1% (n=12/85) increased case confirmation for paucibacillary tuberculosis…” is unclear to me. What is the comparison of “increased” being made to here? Smear microscopy? Microbiological confirmation? Something else? From earlier in the Methods/Results, I thought that everyone in the study had to have a positive sputum, stool, or urine test already – is it the case that some people did not meet this criteria?

**Thank you for bringing this to our attention, we have now clarified the phrases to say *“…providing a 14.1% (n=12/85) increased case confirmation for paucibacillary (“smear-negative”)tuberculosis”* as well as *“…full determination of mutations associated with drug resistance in 14.1% (n=12/85) of patients with paucibacillary tuberculosis.”***

Discussion: Please consider modifying the claim in the discussion that “This study documents that stool-based tNGS improves timely confirmation of tuberculosis and initiation of effective treatment regimens…” There are no results presented here regarding time to results availability or time to treatment initiation.

**Thank you we have revised the sentence to state *“This study documents that stool-based tNGS could provide timely confirmation (<8 hours from sample to drug resistance report) of tuberculosis and initiation of effective treatment regimens in persons affected by paucibacillary tuberculosis, as stool can be obtained at initial diagnosis and has the capability to provide drug resistance predictions.”***

Discussion: Is the group referred to in “Nevertheless, nearly 40% of individuals with undetectable AFB on sputum who qualified for tNGS were persons with undetectable M. tuberculosis culture from sputum, thereby making WGS impossible” the same 34% of individuals who were previously stated as having “undetectable AFB on sputum microscopy” in the Results? If so, I would also include in the Results section that the 34% were also culture-negative, to make it more obvious why they didn’t undergo sputum tNGS.

**This is referring to the 8/21 individuals in the study (38%) who were smear negative and sputum culture negative. This is reported already at the end of the first paragraph in the Results section.**

***“Participant ages ranged from 0 to 68 years (median 32, IQR 15) with 52.4% female (n=11) and 57.1% (n=12) living with HIV. Chest radiography was abnormal (n=19), normal (n=1), and not completed (n=1) and sputum culture was negative in 38% (n=8/21).”***

Conclusion: yes, agree, especially that expanded drug susceptibility testing is a benefit!

**It really is! We are so excited to share this research so tNGS on stool can be used in patients with smear-negative individuals!**

Reviewer #3: This brief report presents the results of a prospective diagnostic accuracy study conducted in Eswatini, a high tuberculosis (TB) burden country, involving participants with negative acid-fast bacilli (AFB) results on sputum microscopy.

Although the World Health Organization (WHO) recommended the use of stool specimens for diagnosing Mycobacterium tuberculosis complex (MTBC) in children using the Cepheid Xpert MTB/RIF assay in 2020—a time frame that coincides with this study—the authors did not reference this guidance in the background, nor in the discussion sections of the report.

**Thank you so much for pointing this out. We have added this in the Background with the appropriate reference with *“…and the WHO has previously endorsed the use of stool for tuberculosis diagnosis in children using the Xpert MTB/Rif test^3^”***

The study includes an image displaying results from phenotypic drug susceptibility testing (pDST), Xpert testing on sputum and stool, and targeted next-generation sequencing (tNGS) on stool using both Nanopore and Deeplex platforms. However, the authors have only analyzed the added diagnostic value of tNGS compared to microscopy. They have not compared the diagnostic yield of stool Xpert with that of tNGS.

**Thank you so much for pointing this out. We had really only focused on the comparison between tNGS on stool and smear microscopy, however you bring up an excellent point about expanding our discussion of the other testing modalities.**

**We have 1) expanded our discussion in Methods on how these other testing modalities were performed with a section on “Other Diagnostic Testing” and 2) provided a descriptive discussion in results comparing tNGS to these other methods.**

***“Other Diagnostic Testing***

***Sputum was evaluated by Xpert Ultra (Cepheid, Sunnyvale, CA, USA) and liquid culture (BACTEC Mycobacteria Growth Indicator Tube [MGIT] system; Becton Dickinson, Franklin Lakes, NJ, USA). Sputum was cultured at the National Tuberculosis Reference Laboratory in Mbabane, Eswatini using MGIT 960 including pDST for rifampin (RIF), isoniazid (INH), and ethambutol (EMB) per national guidelines and in alignment with WHO critical concentrations. When indicated (RIF resistant cases), cultures are also tested for susceptibility to levofloxacin (LFX) and ofloxacin (OFX). Xpert Ultra was used on sputum specimens in accordance with the manufacturer’s instructions; invalid results were repeated, and in case of a second error, were excluded. Stool samples were tested by Xpert Ultra after being processed using the simple one-step stool processing method.^16^”***

Furthermore, rifampicin results and resistance discordances between different specimen types and diagnostic methods have not been assessed. (See attached file)

**Thank you for pointing this out. We have expanded this in our Methods with *“Further analysis was performed with EPI2ME Labs using the ONT automated pipeline ‘wf-tb-amr’ (workflow [v2.0.0-alpha.4], minimap2 [2.24-r1122], samtools [1.18], bedtools [v2.30.0], bcftools [1.18]) with any discrepancies between sequencing and other diagnostic testing additionally run through TB Profiler.^15^”***

***“Rifampin resistance was identified by sputum culture in one case, Xpert Ultra sputum in two cases, and Xpert Ultra stool in one case. Targeted NGS by nanopore identified four cases of rifampin resistance (two cases of His445Asp and two cases of Ser450Leu on the rpoB gene) and Deeplex identified two cases of rifampin resistance (one case of His445Asp and one case of Ser450Leu), identifying resistance found on other diagnostic approaches as well as providing additional identification. One case (patient 9) originally showed rifampin resistance on stool Xpert Ultra but not on nanopore tNGS, however this was identified on TB Profiler with 18x depth of coverage with 100% of reads showing a Ser450Leu mutation in the rpoB region. There were no other discordant determinations of drug resistance or mutations associated with drug resistance between testing modalities; however many cases of drug resistance were identified by one modality with another modality having “no growth” or “no tuberculosis DNA detected.””***

The Authors should address all these issues in revised version
