# Supplementary material for: Screening and targeted sequencing of stool for microbiologic confirmation and drug resistance determination in paucibacillary tuberculosis
Source: PLOS Glob Public Health. 2025 Nov 21;5(11):e0005243. doi: 10.1371/journal.pgph.0005243 (PMC12637971; doi:10.1371/journal.pgph.0005243)
Supplement: Attachment — Submitted filename: Response to Reviewers_Oct25.docx [file pgph.0005243.s003.docx]

Response to Reviewers

**Thank you so much for your invaluable feedback. We have integrated your feedback and suggestions and feel that the manuscript is much stronger. We have provided a detailed response to each of the comments below in bold.**

Reviewer #1: All my comments have been addressed.

**Thank you so much for your assistance with the manuscript!**

Reviewer #2: Thanks to the authors for addressing my comments. I think the flow of information has been strengthened, and the improvement in study population details and findings will certainly be of interest to readers. It is a lot easier to follow now and understand the significance of results. nice work! I have no further substantive comments to make.
Please have a careful read through the paper for grammar and clarity. Please remove contractions and check consistency of terminology.

**Thank you so much! We have checked through the manuscript to correct any small grammar or clarity mistakes.**

**We have removed any contractions and ensured terminology is consistent.**

Reviewer #3: The revised manuscript is well-structured, and the authors have satisfactorily addressed all the reviewers’ comments. However, the manuscript would benefit from the inclusion of a clear figure or Table comparing RIF results across the different diagnostic methods used. This addition would enhance clarity and support the interpretation of findings.

**Thank you so much for your support of this manuscript. We have really appreciated your edits and suggestions and how they have strengthened the manuscript.**

**We acknowledge the importance of communicating the rifampin results to readers, and have added reference to Figure 1 during the discussion of these results, which includes a comparison of RIF results across the different diagnostic methods used. We have also added more information to the key of this figure so it is more apparent which results are RIF.**

**As an additional figure or table on RIF results, specifically, would be redundant and may be confusing to readers (whether it is duplicate from Figure 1 or additional participants) we have not added this, but are happy to do so if all reviewers feel it is necessary and helpful.**
